# Supplementary material for: ID93 + GLA-3M-052-LS vaccine candidate administered by heterologous routes improves BCG efficacy against TB
Source: Front Immunol. 2026 May 1;17:1797767. doi: 10.3389/fimmu.2026.1797767 (PMC13176160; doi:10.3389/fimmu.2026.1797767)
Supplement: Supplementary file 1 [file Table1.docx]

**Supplementary Material**

**Supplementary Tables**

**Table S1. Adjuvant formulation panel.**

| **Adjuvant Name (Dose)** | **Description** | **Immunization Route** | **Reported Immune Response Profile and References** |
| --- | --- | --- | --- |
| GLA-SE (5 µg GLA, 2% v/v squalene) | Synthetic TLR4 agonist in squalene oil-in-water emulsion | i.m. | Serum Abs, systemic Th1 CD4^+^ T cells ^1-3^ |
| 3M-052-Alum (1 µg 3M-052, 100 µg aluminum) | Synthetic TLR7/8 agonist adsorbed to aluminum oxyhydroxide microparticles | i.m. | Serum Abs, long-lived Ab-secreting cells, systemic Th1 CD4^+^ T cells ^4-6^ |
| 3M-052-NanoAlum (1 µg 3M-052, 100 µg aluminum) | Synthetic TLR7/8 agonist adsorbed to aluminum oxyhydroxide nanoparticles stabilized w/poly(acrylic) acid | i.m. | New adjuvant formulation |
| GLA-3M-052-LS (10 µg GLA, 4 µg 3M-052) | Synthetic TLR4 and TLR7/8 agonists in PEGylated liposomes | i.m.or i.n. | Serum and mucosal Abs, long-lived Ab-secreting cells, systemic and mucosal Th1 and Th17 cells^7-9^ |
| Diclofenac-LS (50 µg diclofenac) | Mucosal-associated invariant T cell activator^10, 11^ formulated with cationic liposomes | i.m. | New adjuvant formulation |

**Table S2. Weighting criteria of immune response readouts for desirability index scoring for the adjuvant screening immunogenicity study.**

| **Readout** | **Weight** | **Function** | **Justification** |
| --- | --- | --- | --- |
| IFNγ^+^ CD4^+^ T cells in spleen | 3 | Maximize | Systemic cellular Th1 immunity |
| IFNγ^+^ CD4^+^ T cells in lung | 4 | Maximize | Lung cellular Th1 immunity |
| TNFα^+^ CD4^+^ T cells in spleen | 2 | Maximize | Systemic cellular Th1 immunity |
| TNFα^+^ CD4^+^ T cells in lung | 3 | Maximize | Lung cellular Th1 immunity |
| IL-2^+^ CD4^+^ T cells in spleen | 1 | Maximize | Systemic cellular Th1 immunity |
| IL-2^+^ CD4^+^ T cells in lung | 2 | Maximize | Lung cellular Th1 immunity |
| IL-17^+^ CD4^+^ T cells in spleen | 4 | Maximize | Systemic cellular Th17 immunity |
| IL-17^+^ CD4^+^ T cells in lung | 5 | Maximize | Lung cellular Th17 immunity |
| IFNγ^+^/TNFα^+^/IL-2^+^ CD4^+^ T cells in spleen | 3 | Maximize | Systemic cellular Th1 immunity |
| IFNγ^+^/TNFα^+^/IL-2^+^ CD4^+^ T cells in lung | 4 | Maximize | Lung cellular Th1 immunity |
| IL-10^+^ CD4^+^ T cells in spleen | 1 | Minimize | Undesirable Th2 immune response indicator |
| IFNγ^+^ CD8^+^ T cells in spleen | 2 | Maximize | Effector CD8^+^ T cells in spleen |
| TNFα^+^ CD8^+^ T cells in spleen | 1 | Maximize | Effector CD8^+^ T cells in spleen |
| TNFα^+^ CD8^+^ T cells in lung | 2 | Maximize | Effector CD8^+^ T cells in lung |
| Serum IgG | 4 | Maximize | Serum IgG Ab titers are indicative of systemic immunogenicity |
| IgG2/IgG1 ratio | 4 | Maximize | IgG2c/IgG1 ratio correlates with Th1 immunity |
| IgA in BAL | 5 | Maximize | Mucosal IgA Ab titers are indicative of lung immunogenicity |
| IgG-secreting cells in bone marrow | 4 | Maximize | Long-lived plasma cells are indicator of durable Ab-mediated immunity |

**Table S3. Weighting criteria of immune response readouts for desirability index scoring for the lead candidate immunogenicity study.**

| **Readout** | **Weight** | **Function** | **Justification** |
| --- | --- | --- | --- |
| IFNγ^+^ CD4^+^ T cells in lung | 4 | Maximize | Lung cellular Th1 immunity |
| TNFα^+^ CD4^+^ T cells in lung | 3 | Maximize | Lung cellular Th1 immunity |
| IL-17^+^ CD4^+^ T cells in lung | 5 | Maximize | Lung cellular Th17 immunity |
| IFNγ^+^/TNFα^+^/IL-2^+^ CD4^+^ T cells in lung | 4 | Maximize | Lung cellular Th1 immunity |
| IL-5^+^ CD4^+^ T cells in lung | 1 | Minimize | Undesirable Th2 immune response indicator |
| IFNγ^+^ CD8^+^ T cells in lung | 3 | Maximize | Effector CD8^+^ T cells in lung |
| TNFα^+^ CD8^+^ T cells in lung | 2 | Maximize | Effector CD8^+^ T cells in lung |
| Serum IgG | 4 | Maximize | Serum IgG Ab titers are indicative of systemic immunogenicity |
| IgA in BAL | 5 | Maximize | Mucosal IgA Ab titers are indicative of lung immunogenicity |
| IgG-secreting cells in bone marrow | 4 | Maximize | Long-lived plasma cells are indicator of durable Ab-mediated immunity |

**Supplementary Figures**


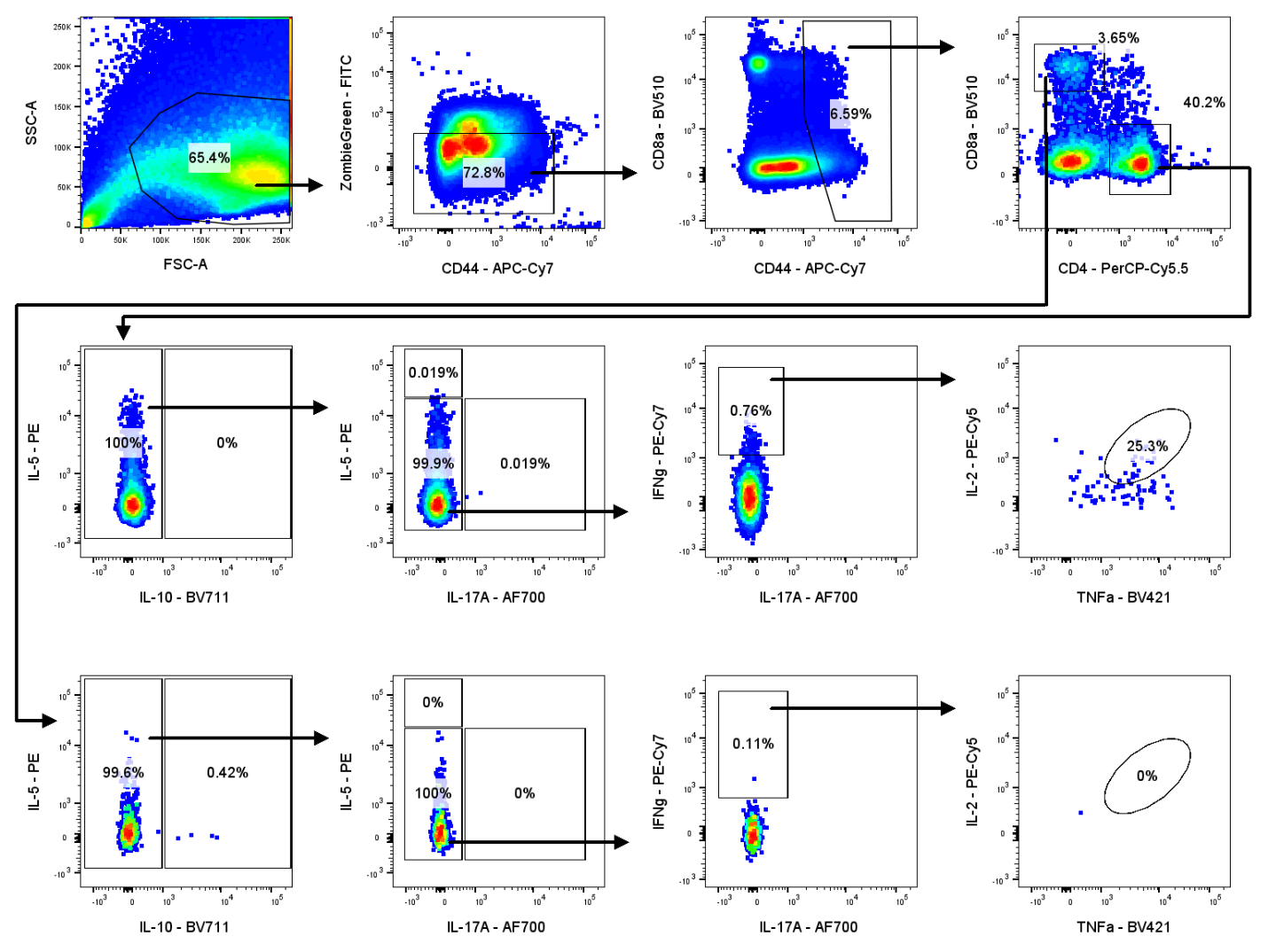


**Figure S1. Spleen flow cytometry gating strategy.** Splenocytes were stained and analyzed via flow cytometry. Cells were first gated on total lymphocytes followed by live cells (FITC^-^) and then activated cells (CD44^+^). Individual gates were drawn for CD4^+^ and CD8^+^ T cells. Within each of these subsets, specific cytokines were gated using biplots, including IL-10, IL-5, IL-17A, IFNγ, IL-2, and TNFα.


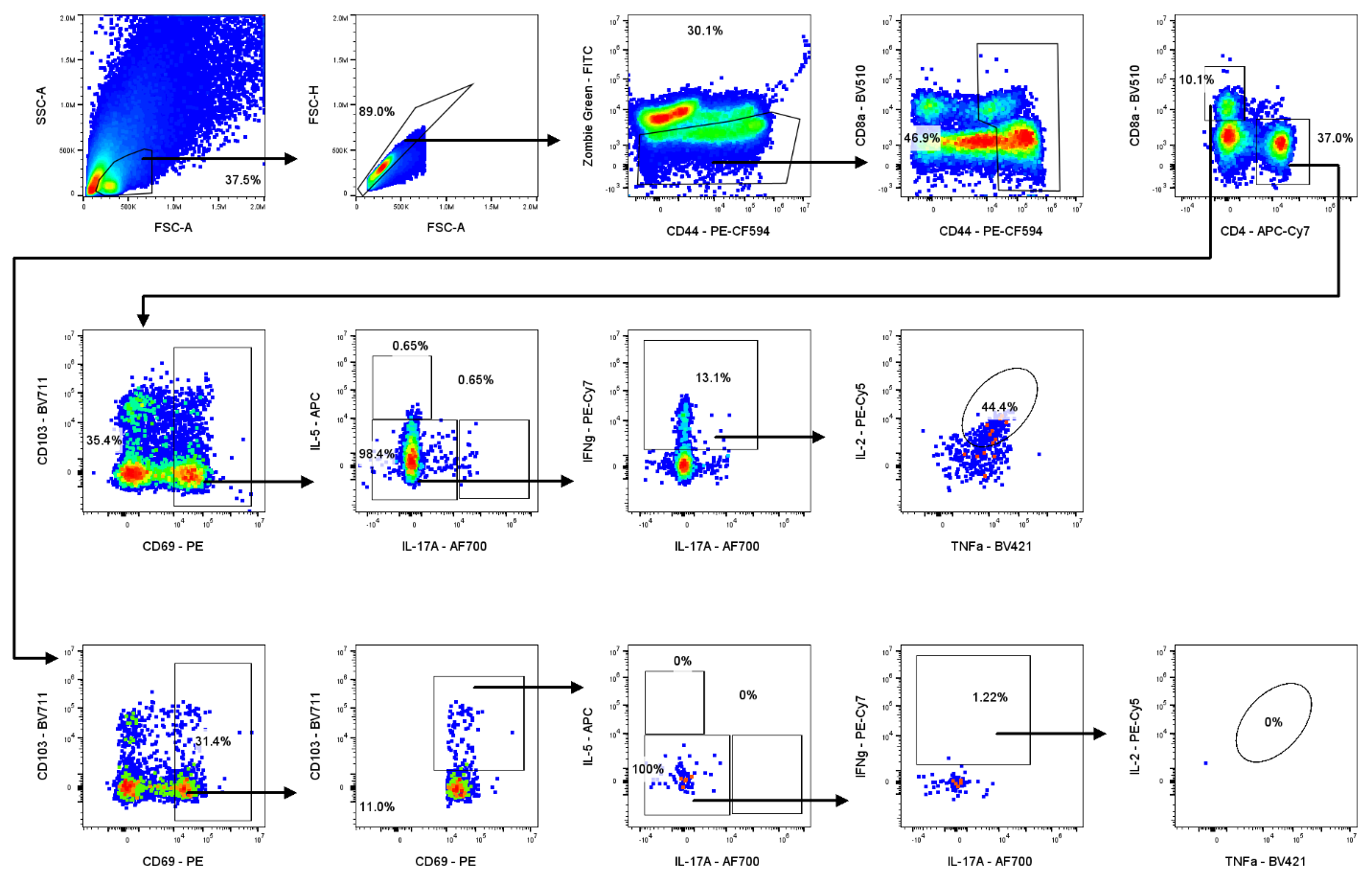


**Figure S2. Lung flow cytometry gating strategy.** Lung cells were stained and analyzed by flow cytometry: First gating on total lymphocytes followed by live cells (FITC^-^) and then activated cells (CD44^+^). Individual gates were drawn for CD4^+^ and CD8^+^ T cells, and further gated for tissue-resident markers (CD69^+^ for both and CD103^+^ for CD8^+^ T cells). Within each of these subsets, specific cytokines were gated using biplots, including IL-5, IL-17A, IFNγ, IL-2, and TNFα.


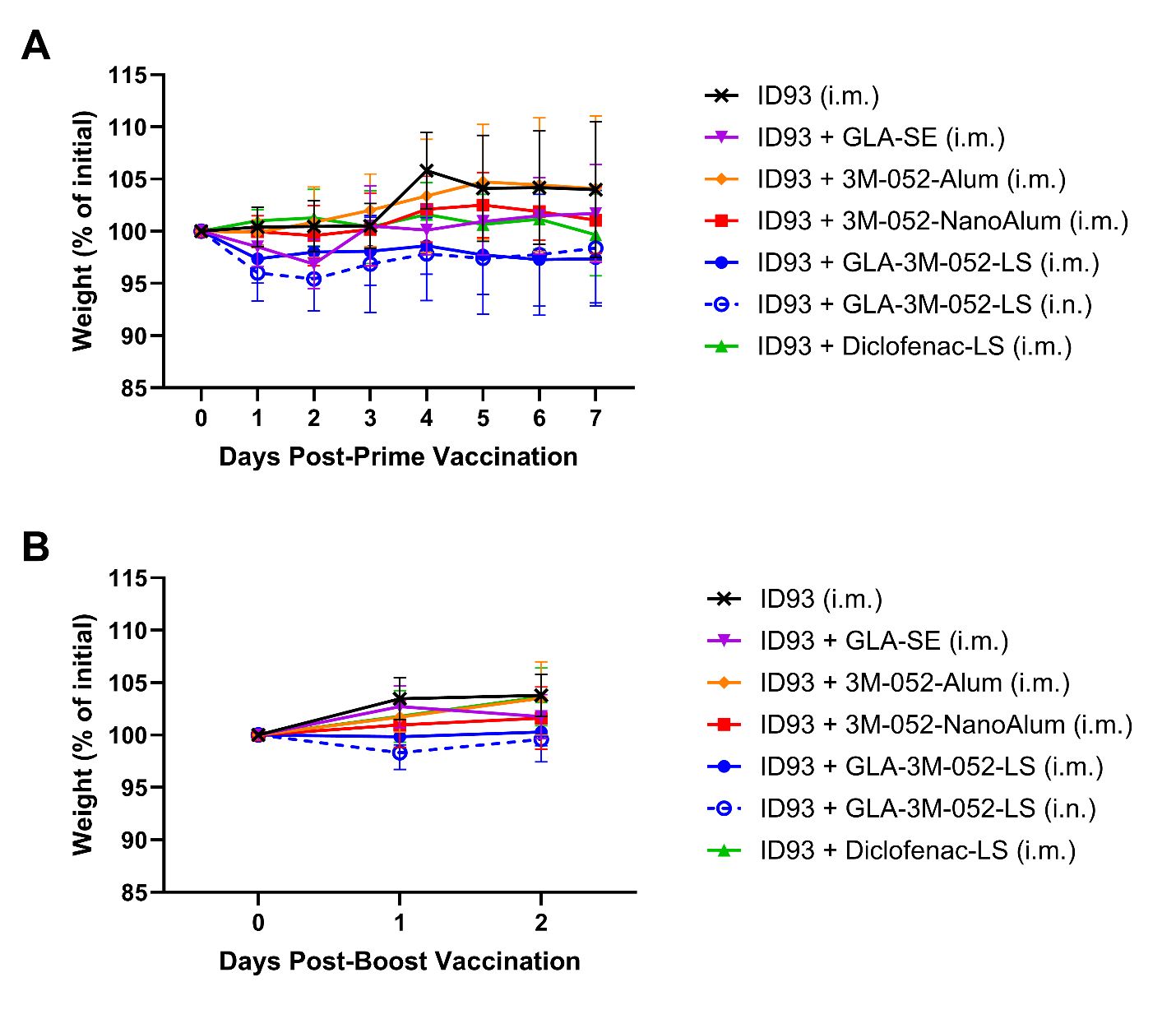


**Figure S3. Mouse weight following immunization.** Change in weight following **(A)** prime immunization and **(B)** boost immunization with the indicated vaccine formulations (*n* = 4-6/group).

**
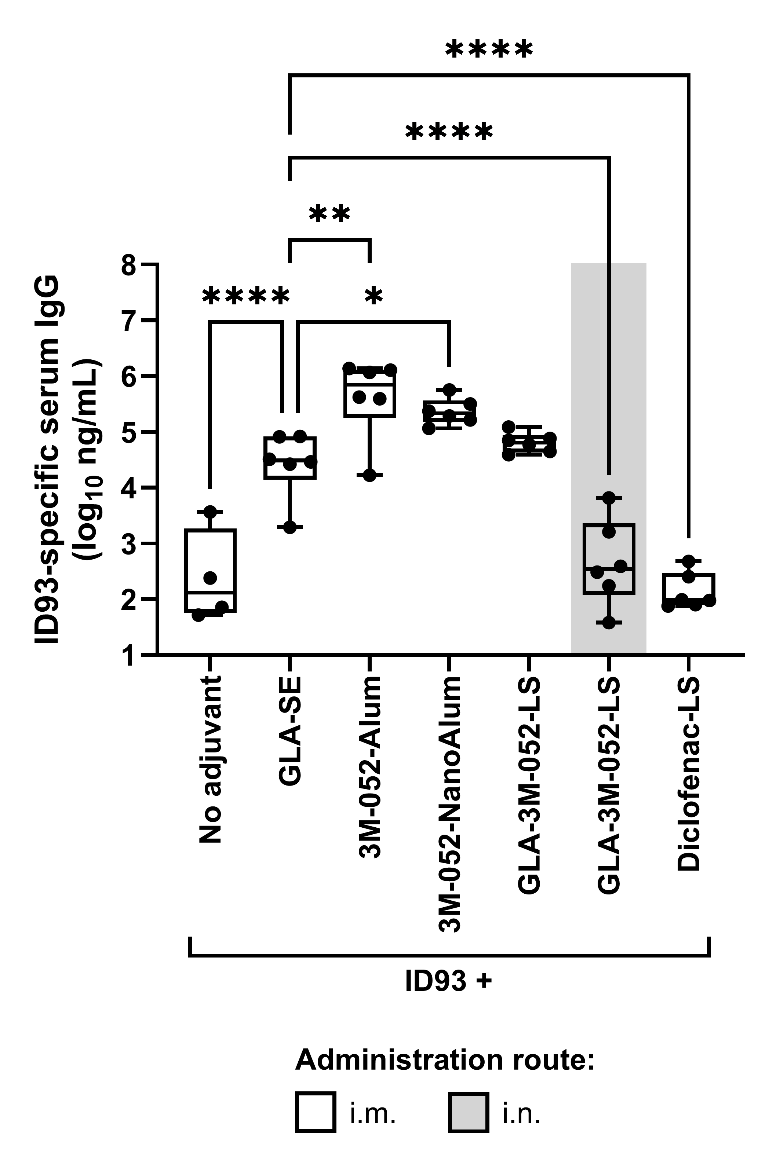
**

**Figure S4. Post-prime antibody immunogenicity elicited by vaccine regimens involving ID93 with distinct adjuvant formulations.** CC004 mice (*n* = 4 to 6/group) were immunized according to the regimens described in Table 1. Three weeks after the first immunization, ID93-specific IgG was measured in the serum. Data were log-transformed and analyzed using one-way ANOVA with Dunnett’s multiple comparisons test; **p* < 0.05, ** *p* < 0.01, **** *p* < 0.0001. Bars indicate median values, boxes indicate the 25-75% spread, and whiskers indicate the minimum and maximum values, with individual data points shown.


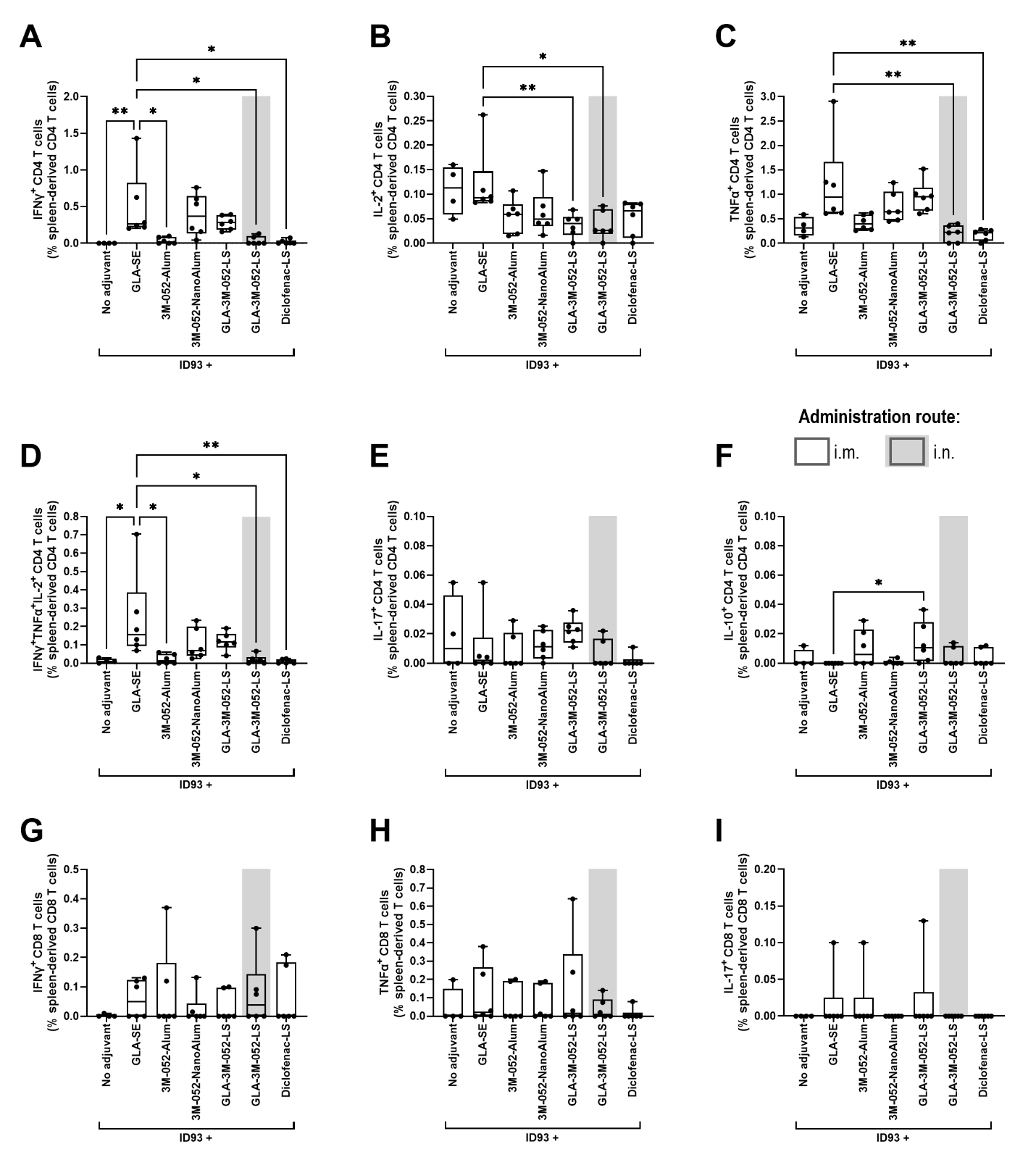


**Figure S5. Cytokines elicited in the spleen by CD4^+^ or CD8^+^ T cells by homologous vaccine regimens involving ID93 with distinct adjuvant formulations.** CC004 mice (*n* = 4 to 6/group) were immunized according to the regimens described in Table 1. One week after the second immunization, ID93-specific immune responses were measured in the spleen. Due to the small group size, the statistical analysis was carried out in the most conservative manner possible, employing the non-parametric Kruskal-Wallis test with Dunn’s correction for multiple comparisons; * *p* < 0.05, ** *p* < 0.01, *** *p* < 0.001. Bars indicate median values, boxes indicate the 25-75% spread, and whiskers indicate the minimum and maximum values, with individual data points shown.

**
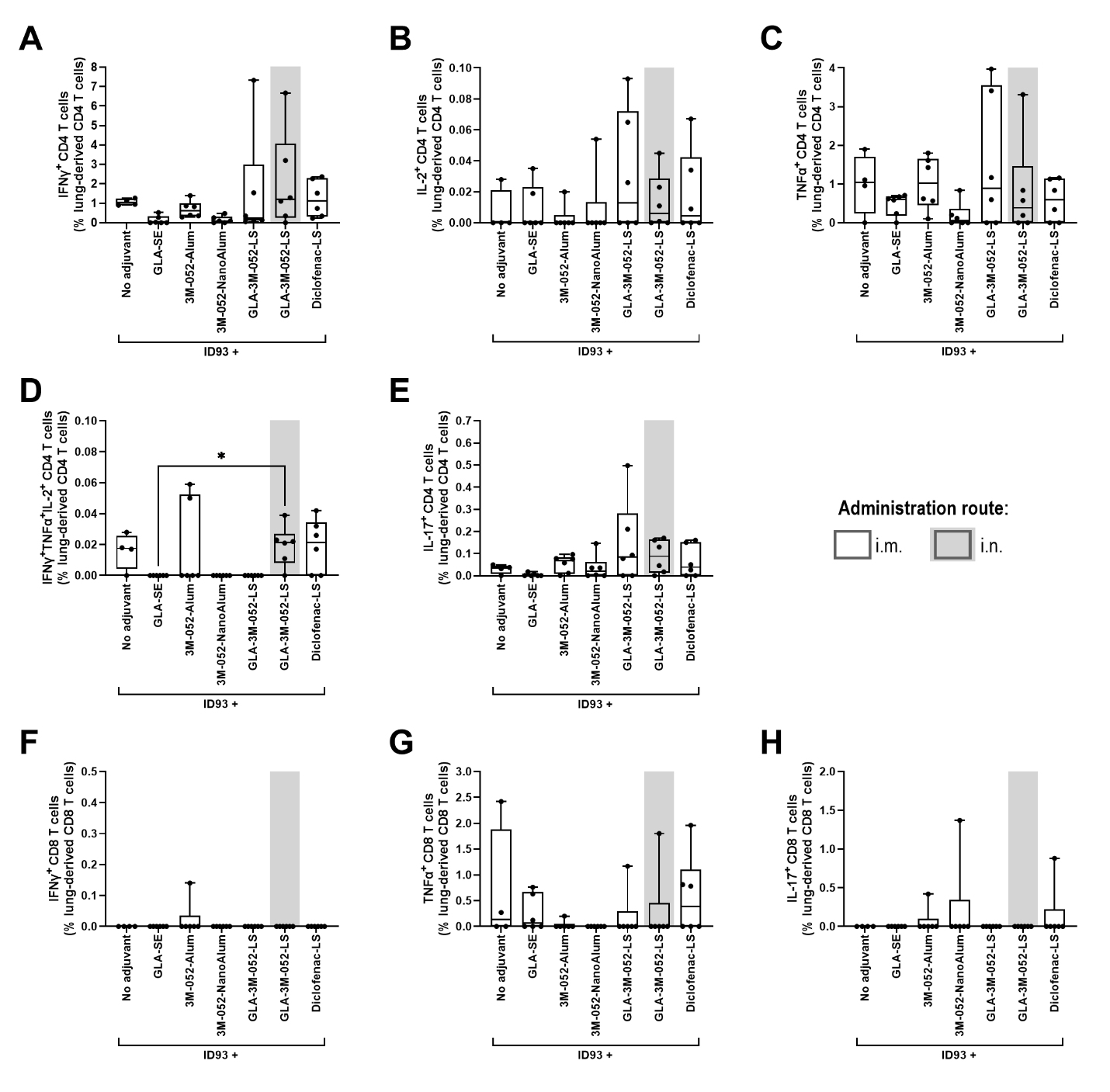
Figure S6. Cytokines elicited in the lung by CD4^+^ or CD8^+^ T cells by homologous vaccine regimens involving ID93 with distinct adjuvant formulations.** CC004 mice (*n* = 4 to 6/group) were immunized according to the regimens described in Table 1. One week after the second immunization, ID93-specific immune responses were measured in the lung. Due to the small group size, the statistical analysis was carried out in the most conservative manner possible, employing the non-parametric Kruskal-Wallis test with Dunn’s correction for multiple comparisons; * *p* < 0.05, ** *p* < 0.01, *** *p* < 0.001. Bars indicate median values, boxes indicate the 25 to 75% spread, and whiskers indicate the minimum and maximum values, with individual data points shown.


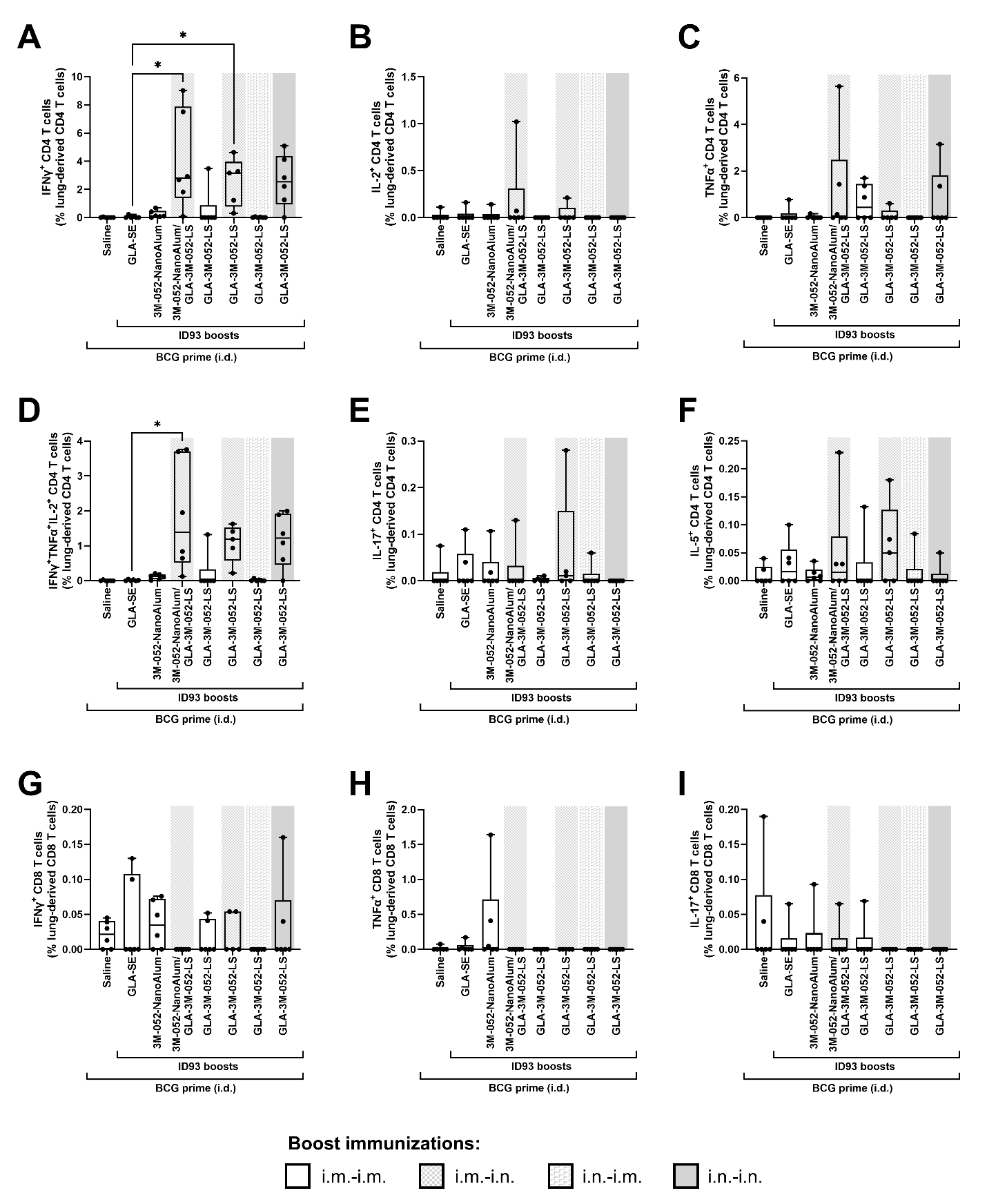


**Figure S7. Cytokines elicited in the lung by CD4^+^ or CD8^+^ T cells by heterologous vaccine regimens involving BCG prime followed by intramuscular or intranasal ID93 boosts with distinct adjuvant formulations.** CC004 mice (*n* = 5 to 6/group) were immunized according to the regimens described in Table 3. Four weeks after the final immunization, ID93-specific immune responses were measured in the lung. Due to the small group size, the statistical analysis was carried out in the most conservative manner possible, employing the non-parametric Kruskal-Wallis test with Dunn’s correction for multiple comparisons; * *p* < 0.05, ** *p* < 0.01, *** *p* < 0.001. Bars indicate median values, boxes indicate the 25 to 75% spread, and whiskers indicate the minimum and maximum values, with individual data points shown.


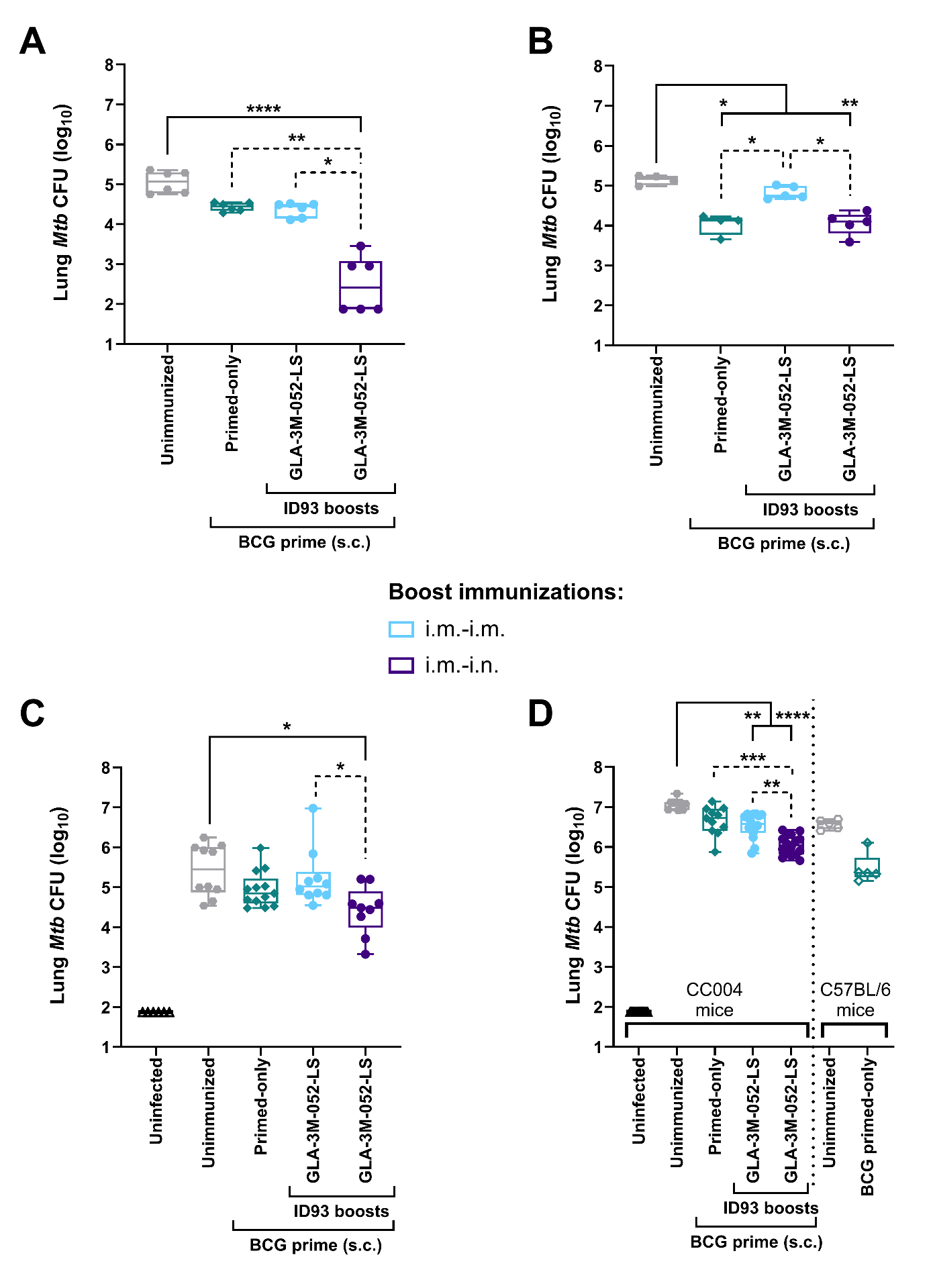


**Figure S8. Separate results from four experiments of lung CFUs in mice immunized by heterologous vaccine regimens involving BCG and ID93 + GLA-3M052-LS.** Lung CFUs (log_10_) from four separate experiments involving the immunization regimens described in Table 5 and additional controls. Combined data are shown in Figure 5. Four weeks after the final immunization, mice were challenged with low-dose aerosolized *Mtb* (25 CFUs for top panels, 100 CFUs for bottom panels). Four weeks following *Mtb* challenge, lungs were harvested and CFUs measured. CFU data were log-transformed and statistical analysis was conducted using the non-parametric Kruskal-Wallis test with Dunn’s correction for multiple comparisons (solid lines: BCG-primed groups compared to unimmunized group; dotted lines: comparison between all BCG-primed groups); *p<0.05, ** p<0.01, *** p<0.001, **** p<0.0001. Box-whisker plots are shown wherein bars indicate median values, boxes indicate the 25 to 75% spread, and whiskers indicate the minimum and maximum values, with individual data points shown.

**
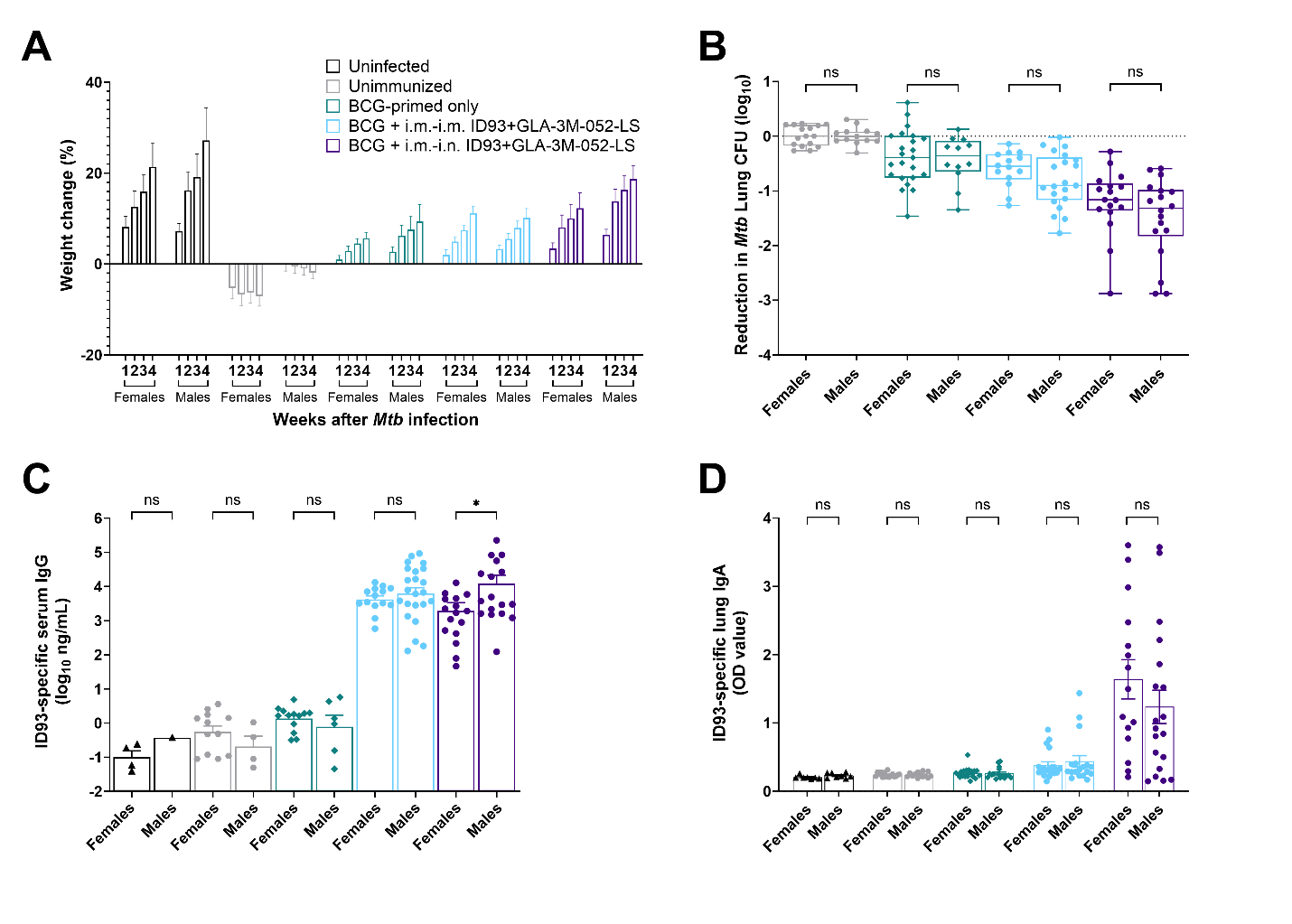
**

**Figure S9. Sex-specific effects in protective efficacy study using heterologous vaccine regimens in BCG-primed and ID93 + GLA-3M-052-LS boosted CC004 mice**. Combined data (total *n*=7 to 20 CC004 mice/sex/group) from the same four separate experiments shown in Supplemental Figure S8 immunized according to the regimens described in Table 5 and Figure 5. Three weeks after the final immunization, mice were challenged with low-dose (25-100 CFUs) aerosolized *Mtb*. Four weeks after *Mtb* challenge, serum and lungs were harvested and lung CFUs were quantified. **(A)** Change in body weight and **(B)** reduction in lung *Mtb* CFUs. CFU data were log-transformed and plotted as reduction in the CFUs (log_10_) compared to the average of unimmunized mice infected with *Mtb*. ID93-specific Abs from **(C)** serum IgG and **(D)** lung IgA. Bars show mean and SEM from four independent experiments. Box-and-whiskers plots show the minimum and maximum with a line at the mean. No consistent significant differences were identified between males and females within each group by unpaired *t-*test pairwise comparisons for CFU reduction, ID93-specific serum IgG, or ID93-specific lung IgA.

REFERENCES

1. Sagawa ZK, Goman C, Frevol A, Blazevic A, Tennant J, Fisher B, Day T, Jackson S, Lemiale F, Toussaint L, Kalisz I, Jiang J, Ondrejcek L, Mohamath R, Vergara J, Lew A, Beckmann AM, Casper C, Hoft DF, Fox CB. Safety and immunogenicity of a thermostable ID93 + GLA-SE tuberculosis vaccine candidate in healthy adults. Nat Commun. 2023;14(1):1138. Epub 20230306. doi: 10.1038/s41467-023-36789-2. PubMed PMID: 36878897; PMCID: PMC9988862.

2. Kramer RM, Archer MC, Orr MT, Dubois Cauwelaert N, Beebe EA, Huang PD, Dowling QM, Schwartz AM, Fedor DM, Vedvick TS, Fox CB. Development of a thermostable nanoemulsion adjuvanted vaccine against tuberculosis using a design-of-experiments approach. Int J Nanomedicine. 2018;13:3689-711. Epub 20180626. doi: 10.2147/IJN.S159839. PubMed PMID: 29983563; PMCID: PMC6028350.

3. Orr MT, Fox CB, Baldwin SL, Sivananthan SJ, Lucas E, Lin S, Phan T, Moon JJ, Vedvick TS, Reed SG, Coler RN. Adjuvant formulation structure and composition are critical for the development of an effective vaccine against tuberculosis. J Control Release. 2013;172(1):190-200. Epub 20130809. doi: 10.1016/j.jconrel.2013.07.030. PubMed PMID: 23933525; PMCID: PMC3871206.

4. Fox CB, Orr MT, Van Hoeven N, Parker SC, Mikasa TJ, Phan T, Beebe EA, Nana GI, Joshi SW, Tomai MA, Elvecrog J, Fouts TR, Reed SG. Adsorption of a synthetic TLR7/8 ligand to aluminum oxyhydroxide for enhanced vaccine adjuvant activity: A formulation approach. J Control Release. 2016;244(Pt A):98-107. Epub 20161112. doi: 10.1016/j.jconrel.2016.11.011. PubMed PMID: 27847326; PMCID: PMC5176129.

5. Phoolcharoen W, Shanmugaraj B, Khorattanakulchai N, Sunyakumthorn P, Pichyangkul S, Taepavarapruk P, Praserthsee W, Malaivijitnond S, Manopwisedjaroen S, Thitithanyanont A, Srisutthisamphan K, Jongkaewwattana A, Tomai M, Fox CB, Taychakhoonavudh S. Preclinical evaluation of immunogenicity, efficacy and safety of a recombinant plant-based SARS-CoV-2 RBD vaccine formulated with 3M-052-Alum adjuvant. Vaccine. 2023;41(17):2781-92. Epub 20230321. doi: 10.1016/j.vaccine.2023.03.027. PubMed PMID: 36963999; PMCID: PMC10027959.

6. Kasturi SP, Rasheed MAU, Havenar-Daughton C, Pham M, Legere T, Sher ZJ, Kovalenkov Y, Gumber S, Huang JY, Gottardo R, Fulp W, Sato A, Sawant S, Stanfield-Oakley S, Yates N, LaBranche C, Alam SM, Tomaras G, Ferrari G, Montefiori D, Wrammert J, Villinger F, Tomai M, Vasilakos J, Fox CB, Reed SG, Haynes BF, Crotty S, Ahmed R, Pulendran B. 3M-052, a synthetic TLR-7/8 agonist, induces durable HIV-1 envelope-specific plasma cells and humoral immunity in nonhuman primates. Sci Immunol. 2020;5(48). doi: 10.1126/sciimmunol.abb1025. PubMed PMID: 32561559; PMCID: PMC8109745.

7. Abhyankar MM, Xu F, Chavez D, Goodroe A, Mendoza E, Chen C, Singh DK, Varnador F, Jr., Sivananthan SJ, Kinsey R, Lykins WR, Murphy BM, Martin AR, Tomai MA, Ghosal S, Casper C, Pedersen K, Petri WA, Jr., Fox CB. Immunogenicity and safety of an Entamoeba histolytica adjuvanted protein vaccine candidate (LecA+GLA-3M-052 liposomes) in rhesus macaques. Hum Vaccin Immunother. 2024;20(1):2374147. Epub 20240801. doi: 10.1080/21645515.2024.2374147. PubMed PMID: 39090779; PMCID: PMC11296537.

8. Abhyankar MM, Orr MT, Kinsey R, Sivananthan S, Nafziger AJ, Oakland DN, Young MK, Farr L, Uddin MJ, Leslie JL, Burgess SL, Liang H, De Lima I, Larson E, Guderian JA, Lin S, Kahn A, Ghosh P, Reed S, Tomai MA, Pedersen K, Petri WA, Jr., Fox CB. Optimizing a Multi-Component Intranasal Entamoeba Histolytica Vaccine Formulation Using a Design of Experiments Strategy. Frontiers in immunology. 2021;12:683157. Epub 20210625. doi: 10.3389/fimmu.2021.683157. PubMed PMID: 34248966; PMCID: PMC8268010.

9. Abhyankar MM, Mann BJ, Sturek JM, Brovero S, Moreau GB, Sengar A, Richardson CM, Agah S, Pomes A, Kasson PM, Tomai MA, Fox CB, Petri WA, Jr. Development of COVID-19 vaccine using a dual Toll-like receptor ligand liposome adjuvant. NPJ Vaccines. 2021;6(1):137. Epub 20211118. doi: 10.1038/s41541-021-00399-0. PubMed PMID: 34795290; PMCID: PMC8602664.

10. Keller AN, Eckle SB, Xu W, Liu L, Hughes VA, Mak JY, Meehan BS, Pediongco T, Birkinshaw RW, Chen Z, Wang H, D'Souza C, Kjer-Nielsen L, Gherardin NA, Godfrey DI, Kostenko L, Corbett AJ, Purcell AW, Fairlie DP, McCluskey J, Rossjohn J. Drugs and drug-like molecules can modulate the function of mucosal-associated invariant T cells. Nat Immunol. 2017;18(4):402-11. Epub 20170206. doi: 10.1038/ni.3679. PubMed PMID: 28166217.

11. Meermeier EW, Harriff MJ, Karamooz E, Lewinsohn DM. MAIT cells and microbial immunity. Immunology and cell biology. 2018;96(6):607-17. Epub 20180309. doi: 10.1111/imcb.12022. PubMed PMID: 29451704; PMCID: PMC6045460.
